# Iron status and risk factors of iron deficiency among pregnant women in Singapore: a cross-sectional study

**DOI:** 10.1186/s12889-019-6736-y

**Published:** 2019-04-11

**Authors:** See Ling Loy, Li Min Lim, Shiao-Yng Chan, Pei Ting Tan, Yen Lin Chee, Phaik Ling Quah, Jerry Kok Yen Chan, Kok Hian Tan, Fabian Yap, Keith M. Godfrey, Lynette Pei-Chi Shek, Mary Foong-Fong Chong, Michael S. Kramer, Yap-Seng Chong, Claudia Chi

**Affiliations:** 1 Department of Reproductive Medicine, KK Women’s and Children’s Hospital, 100 Bukit Timah Road, Singapore, 229899 Singapore; 2 Duke-NUS Medical School, 8 College Road, Singapore, 169857 Singapore; 3 Singapore Institute for Clinical Sciences, Agency for Science, Technology and Research, (A*STAR), 30 Medical Drive, Singapore, 117609 Singapore; 4 Department of Obstetrics & Gynaecology, National University Hospital, 5 Lower Kent Ridge Road, Singapore, 119074 Singapore; 5 Department of Obstetrics & Gynaecology, Yong Loo Lin School of Medicine, National University of Singapore, National University Health System, 1E Kent Ridge Road, Singapore, 119228 Singapore; 6 Department of Haematology-Oncology, National University Cancer Institute, NUH Medical Centre, 5 Lower Kent Ridge Road, Singapore, 119074 Singapore; 7 Department of Maternal Fetal Medicine, KK Women’s and Children’s Hospital, 100 Bukit Timah Road, Singapore, 229899 Singapore; 8 Department of Paediatrics, KK Women’s and Children’s Hospital, 100 Bukit Timah Road, Singapore, 229899 Singapore; 9 Lee Kong Chian School of Medicine, Nanyang Technological University, 11 Mandalay Road, Singapore, 308232 Singapore; 10 Medical Research Council Lifecourse Epidemiology Unit, University of Southampton, Southampton, SO16 6YD UK; 11 National Institute for Health Research Southampton Biomedical Research Centre, University of Southampton and University Hospital Southampton National Health Service Foundation Trust, Southampton, SO16 6YD UK; 12 Department of Paediatrics, Yong Loo Lin School of Medicine, National University of Singapore, 1E Kent Ridge Road, NUHS Tower Block Level 12, Singapore, 119228 Singapore; 13 Khoo Teck Puat-National University Children’s Medical Institute, National University Hospital, National University Health System, 5 Lower Kent Ridge Road, Singapore, 119074 Singapore; 14 Saw Swee Hock School of Public Health, National University of Singapore, 12 Science Drive 2, Singapore, 117549 Singapore; 15 Department of Pediatrics, McGill University Faculty of Medicine, Montreal, QC H3G 2M1 Canada; 16 Department of Epidemiology, Biostatistics and Occupational Health, McGill University Faculty of Medicine, Montreal, QC H3A 1A2 Canada

**Keywords:** Anaemia, Ferritin, Iron status, Pregnancy, Risk factor, Singapore, Soluble transferrin receptor

## Abstract

**Background:**

Iron deficiency is the most prevalent nutrient deficiency and the most common cause of anaemia worldwide. Because of the increased iron requirements during pregnancy, iron deficiency can lead to maternal anaemia and reduced newborn iron stores. We examined the proportion and risk factors of iron deficiency among pregnant women in a developed Asian country.

**Methods:**

Within a prospective cohort in Singapore, 985 Asian women were assessed for iron status at 26–28 weeks’ gestation, with plasma ferritin and soluble transferrin receptor (sTfR) measurements. Iron status was determined according to plasma ferritin concentrations at ≥30 μg/L (iron sufficiency), 15 to < 30 μg/L (modest iron depletion) and < 15 μg/L (severe iron depletion). Multivariable ordinal logistic regression was used to analyze risk factors for modest and severe iron depletion.

**Results:**

The median (25-75th percentile) plasma ferritin concentration was 24.2 (19.9–30.6) μg/L. Overall, 660 (67.0%) and 67 (6.8%) women had modest and severe iron depletion, respectively. Higher plasma sTfR was observed in women with severe iron depletion than among those with iron sufficiency (median 17.6 versus 15.5 nmol/L; *p* < 0.001). Age < 25 years (odds ratio 2.36; 95% confidence interval 1.15–4.84), Malay (2.05; 1.30–3.24) and Indian (1.98; 1.14–3.44) ethnicities (versus Chinese), university qualification (1.64; 1.13–2.38), multiparity (1.73; 1.23–2.44) and lack of iron-containing supplementation (3.37; 1.25–8.53) were associated with increased odds of modest and severe iron depletion.

**Conclusions:**

Nearly three-quarters of Singaporean women were iron deficient in the early third trimester of pregnancy. These results suggest universal screening and supplementation of at-risk pregnancies may be evaluated as a preventive strategy.

**Trial registration:**

NCT01174875. Registered 1 July 2010 (retrospectively registered).

**Electronic supplementary material:**

The online version of this article (10.1186/s12889-019-6736-y) contains supplementary material, which is available to authorized users.

## Background

Iron deficiency is the most prevalent global nutrient deficiency and the most common cause of anaemia worldwide [1, 2]. Iron deficiency represents a spectrum ranging from iron depletion without anaemia (reduced iron stores with a normal haemoglobin (Hb) concentration) to eventual overt anaemia, where the iron supply is insufficient to maintain a normal Hb concentration [3]. Pregnant women are particularly vulnerable to iron deficiency due to substantial increase of iron requirement during pregnancy to support the expansion of erythrocyte mass and plasma volume, and foetal-placental growth [1, 4]. The World Health Organization (WHO) estimates that at least 30–40% of pregnant women are iron deficient and that nearly half are anaemic [5].

For pregnancy, the European Food Safety Authority [6] and the UK Committee on Medical Aspects of Food Policy [7] recommend no increase in iron intake over that for non-pregnant women. The extra iron requirements during pregnancy are considered to be met through cessation of menstrual losses, increased intestinal absorption and mobilisation of maternal iron stores [8]. However, a large proportion of pre-pregnant women or those of reproductive age have low iron stores [9, 10], predisposing them to an increased risk of iron deficiency when becoming pregnant [8]. In Singapore, a developed country, one in five non-pregnant women of reproductive age were found to be anaemic [11].

Either anaemic or non-anaemic iron deficiency prior to and during pregnancy can have adverse consequences for both the mother and offspring, especially with respect to neonatal iron-deficient condition [5, 12]. It was previously thought that neonate was protected from iron deficiency as the developing fetus could acquire sufficient iron from the mother even when she was iron deficient [13]. However, it is now documented that neonatal iron stores can be compromised when the mother is iron deficient or anaemic [13]. Studies on rhesus macaques suggest that infants born to iron deficient mothers before pregnancy had low iron stores after birth [14]. In humans too, newborns from iron deficient mothers at delivery were found to have low iron stores, indicating there is a limited capacity for the fetus to accumulate iron from mothers with low stores [15]. Identifying risk factors for maternal iron deficiency may therefore be helpful in developing preventive strategies to improve offspring health.

Multiple biomarkers have been used to measure iron status. Of these, plasma (or serum) ferritin is the most clinically applicable in pregnancy and has been proposed as the most sensitive single screening test for iron stores [3]. The plasma ferritin threshold used to define iron deficiency in pregnancy varies [16]. According to the World Health Organization [5], plasma ferritin levels of lower than 15 μg/L indicate iron depletion (loss of iron stores) at all stages of pregnancy [3]. This threshold also confirms the presence of iron deficiency anaemia [17]. The Committee for Standards in Haematology from United Kingdom recommends iron supplementation in pregnancy for plasma ferritin less than 30 μg/L [3], a threshold also widely used in clinical practice, including in Singapore, to guide therapy for iron deficiency in pregnancy [16]. This threshold has higher specificity than the lower threshold or when compared with other pregnancy biomarkers [18].

Although iron deficiency anaemia in pregnancy has been a frequent focus of research, few studies have investigated iron deficiency per se in pregnancy [10], leading to uncertainty about its clinical and public health significance. This is particularly true for women living in relatively affluent settings. In this study in highly developed Singapore, we aimed 1) to examine the proportion of iron deficiency in women during the early third trimester of pregnancy, and 2) to assess risk factors associated with iron deficiency in pregnancy.

## Methods

### Study design and participants

Data were drawn from the Growing Up in Singapore Towards healthy Outcomes (GUSTO) pregnancy cohort study (www.clinicaltrials.gov, NCT01174875), detailed elsewhere [19]. This study was conducted according to the guidelines laid down in the Declaration of Helsinki. Ethical approval was obtained from the Domain Specific Review Board of Singapore National Healthcare Group (reference D/09/021) and the Centralised Institutional Review Board of SingHealth (reference 2009/280/D).

Pregnant women attending antenatal visits (< 14 weeks’ gestation) in KK Women’s and Children’s Hospital (KKH) and National University Hospital (NUH) were recruited into the GUSTO study between June 2009 and September 2010. KKH and NUH are the two major public maternity units in Singapore. Recruited women were Singapore citizens or permanent residents between 18 and 50 years of age with biparentally homogeneous ethnicity (Chinese, Malay or Indian). Those who conceived naturally were included in this study. Women receiving chemotherapy or psychotropic drugs and those with type 1 diabetes mellitus were excluded. Informed written consent was obtained from all women prior to recruitment.

### Data collection

Recruited women returned to the hospitals at 26–28 weeks’ gestation for a follow-up study visit. Detailed interviews were conducted in the clinics by trained staff. Data on maternal socio-demographics, educational attainment, obstetric history, smoking status, iron-containing supplementation and anaemia history were collected. Women were asked about the highest level of education attained. Number of previous pregnancies and their outcomes were recorded to determine parity, which included all live- and stillbirths occurring at or after 24 weeks’ gestation, to classify women as nulliparous or parous. Positive smoking status was defined as any cigarette smoking in the current pregnancy. Data on iron-containing supplements, including those taken as part of a multivitamin and mineral supplement or prenatal supplement, was recorded if it was taken for more than once a week in the current pregnancy. Women were asked if they had any history of anaemia in previous pregnancies, either antenatally or post-partum. Data on maternal Hb concentration (g/dl) in early pregnancy (14 weeks’ gestation or less) was collected from the hospital medical records. Women were classified as anaemic if their Hb was less than 11 g/dL [5].

### Anthropometry

Self-reported pre-pregnancy weight and measured booking weight at the first antenatal clinic visit (≤14 weeks’ gestation) were recorded. Height was measured with a portable stadiometer (Seca 213, Hamburg, Germany) at 26–28 weeks gestation. Body mass index (BMI) was determined using the formula of weight (kg)/ height (m^2^). Since the early pregnancy BMI obtained at the first clinic visit was strongly correlated with self-reported pre-pregnancy BMI (r = 0.96, *p* < 0.001), was free from recall bias and had a lower percentage of missing values than pre-pregnancy BMI (5.7% vs. 7.7%), it was used for all study analyses. BMI status was categorized as < 23 versus ≥23 kg/m^2^ based on cut-off points for Asian populations [20].

### Plasma ferritin and soluble transferrin receptor assessments

At 26–28 weeks’ gestation, maternal fasting blood samples were collected for the measurements of plasma ferritin and soluble transferrin receptor (sTfR). Plasma ferritin (μg/L) was measured using the sandwich enzyme-linked immunosorbent assay (ELISA) method (AssayMax Human Ferritin ELISA, AssayPro, United States) with an intra-assay coefficient of variation (CV) of 2.9%. The kit standard (AssayMax Human Ferritin Standard) was used as a control, which has been calibrated against WHO International Standard. Women were classified as having iron sufficiency, modest iron depletion and severe iron depletion based on plasma ferritin concentrations of ≥30, 15 to < 30 and < 15 μg/L, respectively [3, 5]. Both modest and severe iron depletion were defined as iron deficiency. Since ferritin is an acute phase protein whose concentration can increase markedly during infection and other inflammatory conditions [16], we quantified the levels of sTfR as an additional biomarker of iron deficiency, since it is considered to be less affected by acute-phase reactants [3, 21]. Elevated sTfR indicates the presence of functional iron deficiency. Plasma sTfR (nmol/L) was measured using an ELISA (Human sTFr ELISA, BioVendor, Czech Republic) with an intra- and inter-assay CV of 10.9 and 4.8%, respectively. Control human serum samples (BioVendor Quality Control) were run in each assay as an internal control.

### Statistical analysis

Descriptive statistics are presented as percentages for categorical variables; means, standard deviations, medians and 25-75th percentiles for continuous variables. Comparisons of demographics and characteristics between women with iron sufficiency, iron depletion and severe iron depletion were performed using Fisher’s exact tests for categorical variables, ANOVA or Kruskal-Wallis tests for continuous variables. Spearman correlation was used to analyse the continuous association between maternal Hb in early pregnancy and plasma ferritin at 26–28 weeks’ gestation.

Ordinal logistic regression with three ordinal levels was performed for multivariable analyses to assess independent risk factors for iron depletion and severe iron depletion. Compared to a series of binary logistic regression or using multinomial logistic regression, the use of an ordinal logistic regression model helps to increase the power by making full use of the structure of an ordinal scale, producing a more stable estimate and summary with a broad interpretation, applicable across multiple dichotomizations of outcome [22]*.* In determining variables to be included or excluded from the multivariable model, it has been shown that methods using pre-determined *p*-value criteria in the univariate analysis are inappropriate, and likewise for automated variable selection procedures (e.g. forward, stepwise) [23]. This is because confounding effects and inter-correlations between independent variables are not being considered, which can lead to biased and distorted outcomes [23]. A better way to determine which variables should be included in the multivariable model is by using clinical judgement [23], as done in other studies identifying risk factors of an outcome [24, 25]. In this analysis, we selected the potential risk factors and built the model based on a literature review [26–28], clinical knowledge and by using a directed acyclic graph. In multivariable ordinal logistic regression analysis, we entered the following potential risk factors simultaneously into the model: maternal age (< 25, 25–34 or ≥ 35 years), BMI (< 23 or ≥ 23 kg/m^2^), ethnicity (Chinese, Malay or Indian), education (below or at university levels), parity (nulliparous or multiparous), smoking status (no or yes), iron-containing supplementation (no or yes) and history of anaemia (no or yes). The fit of model and proportional odds assumption were checked and met. The proportional odds ratio as presented in this study could be viewed as independent from the degree of severity used to classify the iron status and was thus, valid over all cut-points simultaneously.

Missing values for maternal BMI (*n* = 6), education (*n* = 13), parity (*n* = 1), smoking status (*n* = 2), iron-containing supplementation (*n* = 97) and history of anaemia (*n* = 1) were imputed 100 times using multiple imputation analyses by chained equations. The results of the 100 analyses were pooled using Rubin’s rule. Complete-case analysis was performed as a sensitivity analysis (*n* = 871). All statistical analyses were two-sided with a 5% significance level and were performed using SPSS software, Version 20 (USA).

## Results

**Fig. 1:**
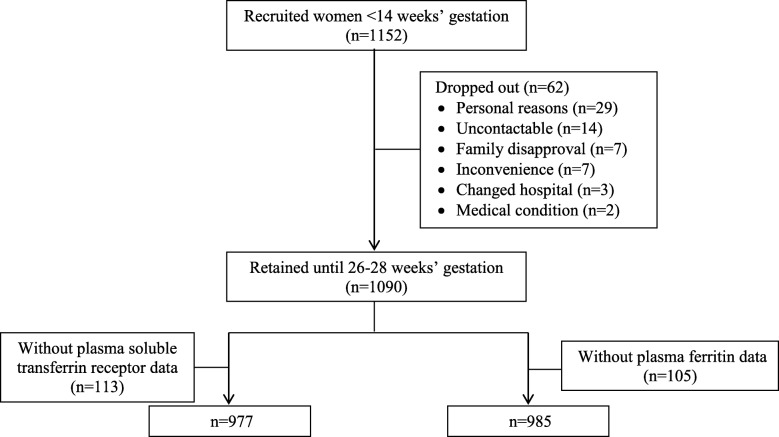
Flow chart of the study

Of 1152 recruited pregnant women who conceived naturally, 1090 (94.6%) of them returned for a study visit at 26–28 weeks’ gestation. A total of 977 (89.6%) women had sufficient plasma for analysis of sTfR concentrations; while 985 (90.4%) women had sufficient plasma for analysis of ferritin concentrations and were included in the present analyses (Fig. 1). Compared to excluded women, those included were more likely to have age greater than 35 years (21.9 versus 13.1%; *P* < 0.001) and attained university education (33.7 versus 22.8%; *P* = 0.003). They were, however, similar in every other factor assessed, including BMI status, ethnicity, parity, smoking status, iron-containing supplementation and history of anaemia (see Additional file 1: Table S1).

Table 1 shows the demographics and characteristics of women categorized by iron status. Overall (*n* = 985), 73.8% (*n* = 727) of pregnant women were iron deficient (ferritin < 30 μg/L). Among these women, the majority had modest iron depletion (ferritin 15 to < 30 μg/L, *n* = 660; 67.0%), while 6.8% (*n* = 67) had severe iron depletion (ferritin < 15 μg/L). Compared to women with iron sufficiency (ferritin ≥30 μg/L), those with modest iron depletion were more likely to belong to the Malay (27.6 versus 21.7%; *P* < 0.001) or Indian (21.8 versus 13.6%; *P* < 0.001) ethnic groups, to attain university education (36.5 versus 29.5%; *P* = 0.043), to be multiparous (60.0 versus 51.2%; *P* = 0.013), less likely to have taken iron-containing supplements during pregnancy (85.5 versus 93.4%; *P* = 0.001) and had higher plasma sTfR concentration (median 16.7 versus 15.5 nmol/L; *P* < 0.001). Out of 422 pregnant women with available information on Hb in early pregnancy, 81 (19.2%) had anaemia, 91% of whom had iron deficiency (modest and severe iron depletion) at 26–28 weeks. Among non-anaemic women in early pregnancy (*n* = 341), 255 (74.8%) were iron deficient at 26–28 weeks. A significant correlation was observed between Hb in early pregnancy and plasma ferritin at 26–28 weeks’ gestation (r = 0.22, *p* < 0.001).

**Table 1:** Maternal characteristics by iron status based on plasma ferritin concentrations in the GUSTO study (*n* = 985)

Characteristics	Total (*n* = 985)	Iron sufficiency (*n* = 258; 26.2%)	Modest iron depletion (*n* = 660; 67.0%)	Severe iron depletion (*n* = 67; 6.8%)	p^a^
Age, %					0.044
< 25 years	12.8	10.1	12.9	22.4	
25–34 years	65.3	64.0	66.2	61.2	
≥ 35 years	21.9	26.0	20.9	16.4	
Body mass index, kg/m^2^					0.410
Mean	23.6	23.6	23.6	24.4	
Standard deviation	4.8	4.7	4.7	5.2	
Body mass index, %					0.727
< 23 kg/m^2^	53.9	54.7	54.1	49.3	
≥ 23 kg/m^2^	46.1	45.3	45.9	50.7	
Ethnicity, %					< 0.001
Chinese	54.4	64.7	50.6	52.2	
Malay	26.6	21.7	27.6	35.8	
Indian	19.0	13.6	21.8	12.0	
Education, %					0.016
None/Primary/Secondary	66.3	70.5	63.5	77.6	
University	33.7	29.5	36.5	22.4	
Parity, %					0.046
Nulliparous	42.5	48.8	40.0	43.3	
Multiparous	57.5	51.2	60.0	56.7	
Smoking status, %					0.751
No	97.5	96.9	97.7	97.0	
Yes	2.5	3.1	2.3	3.0	
Iron-containing supplementation, %					0.003
No	12.5	6.6	14.5	14.9	
Yes	87.5	93.4	85.5	85.1	
History of anaemia, %					0.952
No	94.8	95.0	94.7	95.5	
Yes	5.2	5.0	5.3	4.5	
Hemoglobin at early pregnancy^b^ g/dL					0.010
Median	11.9	12.3	11.8	11.8	
Interquartile range	11.2–12.6	11.6–13.0	11.0–12.6	10.1–12.4	
Anaemia at early pregnancy^b^ %					0.005
No	80.8	92.5	77.9	74.2	
Yes	19.2	7.5	22.1	25.8	
Plasma ferritin, μg/L					< 0.001
Median	24.2	36.2	22.6	12.3	
Interquartile range	19.9–30.6	32.3–42.1	19.7–25.6	9.4–13.9	
Plasma soluble transferrin receptor,^c^ nmol/L					< 0.001
Median	16.4	15.5	16.7	17.6	
Interquartile range	14.3–19.7	13.6–18.2	14.4–20.0	15.0–21.6	

*GUSTO* Growing Up in Singapore Towards healthy Outcomes. Values are presented as percentages for categorical variables, and median (interquartile range) for continuous variables. Iron sufficiency, modest and severe iron depletion are defined as having plasma ferritin concentrations of ≥30 μg/L, 15 to < 30 μg/L and < 15 μg/L, respectively

^a^*p* values for differences between groups are obtained from Fisher’s exact tests for categorical variables, ANOVA or Kruskal-Wallis tests for continuous variables

^b^Data on hemoglobin concentrations and anemia at early pregnancy are only available for subsample of women, *n* = 422

^c^Data on plasma soluble transferrin receptor are only available for 977 women

Table 2 shows the results of the univariable and multivariable ordinal logistic regression analyses. In the multivariable model, which was of good fit, maternal age < 25 years (OR 2.36; 95% CI 1.15, 4.84), Malay (OR 2.05; 95% CI 1.30, 3.24) and Indian (OR 1.98; 95% CI 1.14, 3.44) ethnicities, university qualification (OR 1.64; 95% CI 1.13, 2.38), multiparity (OR 1.73; 95% CI 1.23, 2.44) and lack of iron-containing supplementation during pregnancy (OR 3.37; 95% CI 1.25, 8.53) were associated with a significantly increased odds of modest and severe iron depletion. In the sensitivity analysis based on women with complete data (*n* = 871), the results remained similar (see Additional file 2: Table S2).

**Table 2:** Factors associated with iron status during pregnancy assessed by ordinal logistic regression analyses (*n* = 985)

Characteristics	Univariable			Multivariable		
	Ordinal OR^a^	95% CI	*P*	Ordinal OR^a^	95% CI	*P*
Age						
< 25 years	2.00	0.99, 4.01	0.050	2.36	1.15, 4.84	0.019
25–34 years	1.31	0.93, 1.86	0.126	1.26	0.88, 1.80	0.209
≥ 35 years	Reference			Reference		
Body mass index						
< 23 kg/m^2^	Reference			Reference		
≥ 23 kg/m^2^	1.04	0.75, 1.44	0.818	0.90	0.65, 1.26	0.551
Ethnicity						
Chinese	Reference			Reference		
Malay	1.83	1.17, 2.85	0.008	2.05	1.30, 3.24	0.002
Indian	2.17	1.22, 3.85	0.008	1.98	1.14, 3.44	0.015
Education						
None/ Primary/ Secondary	Reference			Reference		
University	1.36	0.93, 1.97	0.110	1.64	1.13, 2.38	0.010
Parity						
Nulliparous	Reference			Reference		
Multiparous	1.48	1.07, 2.05	0.016	1.73	1.23, 2.44	0.002
Smoking status						
No	Reference			Reference		
Yes	0.73	0.32, 1.65	0.449	0.62	0.27, 1.40	0.250
Iron-containing supplementation						
Yes	Reference			Reference		
No	3.46	1.23, 9.74	0.019	3.37	1.25, 8.53	0.016
History of anemia						
No	Reference			Reference		
Yes	1.05	0.50, 2.22	0.900	0.95	0.44, 2.04	0.893

*OR* Odds ratio, *CI* confidence interval. Iron status was defined according to three ordinal categories of plasma ferritin concentrations: ≥30 μg/L (iron sufficiency), 15 to < 30 μg/L (modest iron depletion) and < 15 μg/L (severe iron depletion)

^a^Proportional odds ratios with iron sufficiency as the base level, for severe iron depletion versus modest iron depletion/ iron sufficiency, and severe/ modest iron depletion versus iron sufficiency

## Discussion

During pregnancy, iron status tends to decline with advancing gestation, which can be due to iron mobilization or to haemodilution that peaks at 24–26 weeks [12]. Therefore, different cut-offs to define iron deficiency across gestation have been suggested [10]. Using stained bone marrow aspirates as the gold standard to evaluate iron status among pregnant women, most of those with an absence of stainable bone marrow haemosiderin iron have plasma ferritin < 30 μg/L in the second and third trimesters of pregnancy [18]. In the present study, at 26–28 weeks of gestation, only a quarter of pregnant women had sufficient iron stores, as defined by a plasma ferritin of at least 30 μg/L. The majority of women (74%) in our developed country was found to be iron deficient (ferritin < 30 μg/L), in which 67% had modest iron depletion (ferritin 15 to < 30 μg/L) and 7% had severe iron depletion (ferritin < 15 μg/L). Women with age < 25 years, Malay or Indian ethnicity, attained university qualification, multiparity and those not using iron-containing supplements during pregnancy were more likely to be iron depleted.

The prevalence of iron deficiency in pregnant women varies worldwide [28–31], depending on the definition used, study population, gestational age at assessment and assay method. Based on a plasma ferritin threshold of < 30 μg/L, the proportion of iron deficiency among women at 26–28 weeks in this study (74%) was higher than that reported among pregnant women in Portugal at early pregnancy (38%) [31], but lower than that in pregnant women in Scotland at late pregnancy (90%) [29]. This is consistent with the notion that ferritin concentrations decline with the progression of pregnancy [32, 33]. These variations may simply reflect normal pregnancy physiology, rather than differences among populations. Though the proportion of our study women with severe iron depletion (ferritin < 15 μg/L; 7%) was much lower than rates reported among pregnant women in other developed countries [10], a high proportion of our women had modest iron depletion (ferritin 15 to < 30 μg/L). This high number warrants further investigation for subsequent biochemical and clinical consequences among both the mother and offspring.

We found that one in five women reported having been anaemic in the first trimester, similar to the prevalence of anaemia (22.2%) in women of reproductive age in Singapore [11]. The vast majority of these women (91%) were found to be iron deficient at 26–28 weeks despite reported iron-containing supplementation (72%). Since nearly 80% of anaemia in the Singapore pregnant population is due to iron deficiency [26], it is likely that most anaemic women in our study already had iron deficiency anaemia in early pregnancy, and even prior to conception. In view that anaemia is a late manifestation of iron deficiency, our findings are not surprising. Of interest is that almost just as high a proportion (75%) of non-anaemic women in early pregnancy was also found subsequently to be iron deficient at 26–28 weeks. Evaluation of *mean corpuscular volume (*MCV) and mean corpuscular haemoglobin (MCH) together with ferritin may be required in addition to Hb levels in the screening of women at the beginning of pregnancy to better detect iron deficiency and initiate appropriate iron treatment. Assessment of thalassemia, estimated at 9% in this population [34], should be considered during the screening procedure as the disorder might lead to abnormalities in the levels of red blood cell indices and biomarkers for iron status. Meanwhile, the role of prophylactic iron supplementation before the onset of iron deficiency anaemia remains controversial, given the reported associations of universal iron supplementation with development of gestational diabetes [35] and pregnancy-induced hypertension [36].

Similar to a local Singapore study in non-pregnant women [37], Malay and Indian pregnant women were more likely to have iron deficiency than Chinese pregnant women. Differences in dietary practice and/ or iron absorption ability may contribute to the higher proportions of iron deficiency in Malays and Indians [37]. Among the ethnic groups, Chinese women have been reported to consume more meat, poultry and egg, contributing to a rich source of haem-iron with a higher bioavailability; Malay women consumed fewer fruits and vegetables and had a lower vitamin C intake, which could reduce iron absorption; while Indian women tended to be vegetarian where the absorption of non-haem might be inhibited by phytates present in vegetables and cereals [37, 38]. Younger age may reflect poorer nutrition with reduced dietary iron intake, while multiparity may reflect depleted iron supply with increasing number of pregnancies [28, 32]. The finding of a positive association between education level and iron deficiency is supported by previous reports [27, 39], suggesting that knowledge, understanding or awareness of iron deficiency does not translate into action to increase iron-rich food intake or the use of iron supplements. Despite a high reported use of iron-containing supplements among our pregnant women (88%), a large proportion of them had iron deficiency at 26–28 weeks. The frequency and dosage consumed and compliance with iron-containing supplement intake were not recorded, moreover, which have been reported to influence iron status [10]. Otherwise, the absorption of iron from these iron-containing tablets is probably low due to the absorptive interaction of iron with other divalent metal ions in the tablets (e.g. zinc, manganese, calcium) [40].

We acknowledge several limitations. Variability in plasma volume expansion can affect the interpretation of biomarker levels, including levels of plasma ferritin, particularly late in pregnancy. The best time to detect maternal iron deficiency has been suggested to be in early pregnancy, before the plasma volume is fully expanded. As we did not measure plasma ferritin in early pregnancy, the present findings should therefore be interpreted cautiously. However, the use of a plasma ferritin cut-off of < 30 μg/L to define iron deficiency in the late second trimester is supported by van den Broek and colleagues [18], using bone marrow iron as a reference among pregnant women at mid and late pregnancy. Notwithstanding this, our findings probably include a mix of both physiologic plasma volume expansion and true iron deficiency. Multiple indicators of iron status such as transferrin saturation and hepcidin could be collectively used for better determination of iron status at later stages of pregnancy. Data on iron-containing supplementation was self-reported and therefore subject to recall errors. Data on dietary factors that may affect iron absorption were not available in this study. Measurement of Hb level at 26–28 weeks’ gestation is not part of routine clinical assessment in this setting, so data on maternal anaemia status was not available at the same time point as the plasma ferritin measurement. However, the use of a plasma ferritin cut-off of < 15 μg/L has been documented as an indicator for iron deficiency anaemia [17]. The proportions of women who reported smoking and a history of anaemia were small (≤5%) and insufficient to provide reliable conclusions regarding these groups. Some differences in characteristics (i.e., age and education) were noted between included and excluded women, which might have introduced selection bias. Finally, the lack of data on maternal inflammatory biomarkers (e.g. C-reactive protein and α-1-acid glycoprotein) is another limitation. Though sTfR has previously thought to be less affected by inflammation, a recent study found that sTfR has a weak but consistent relation with α-1-acid glycoprotein concentrations [41]. Ferritin concentrations are widely recognised to increase in the presence of inflammation [16] and pregnancy has been associated with low-grade inflammation [42]; thus the detection rate of iron deficiency in this study may be underestimated with no adjustment for inflammatory markers [43].

## Conclusions

In conclusion, a substantial proportion of Asian pregnant women were found to be iron deficient at 26–28 weeks in Singapore, highlighting the potential importance of routine monitoring and screening for iron deficiency at several time points during pregnancy for timely commencement of iron treatment. Concerted efforts, including routine dietary advice (e.g. consuming plenty of iron-rich foods with a higher iron bioavailability, along with items containing vitamin C) and individual iron supplementation prophylaxis before and during pregnancy should be considered in this population for optimal maternal and offspring health. However, more research is needed to determine the appropriate use and dose of iron supplements for differing degrees of iron deficiency in pregnancy. Although the treatment of iron deficiency anaemia is not disputed, the maternal and offspring benefits of iron supplementation in iron deficiency with the absence of anaemia require rigorous evaluation in randomized trials.

## Additional files

Additional file 1: **Table S1.** Comparison of characteristics between excluded and included participants (*n* = 1152). (DOC 63 kb)

Additional file 2: **Table S2.** Factors associated with iron status during pregnancy assessed by ordinal logistic regression analyses (*n* = 871). (DOC 63 kb)

## Abbreviations

BMI: Body mass index
ELISA: Enzyme-linked immunosorbent assay
GUSTO: Growing Up in Singapore Towards healthy Outcomes
Hb: Haemoglobin
KKH: KK Women’s and Children’s Hospital
MCH: Mean corpuscular haemoglobin
MCV: Mean corpuscular volume
NUH: National University Hospital
sTfR: Soluble transferrin receptor
WHO: World Health Organization
