# Supplementary material for: Iron status and risk factors of iron deficiency among pregnant women in Singapore: a cross-sectional study
Source: BMC Public Health. 2019 Apr 11;19:397. doi: 10.1186/s12889-019-6736-y (PMC6460529; doi:10.1186/s12889-019-6736-y)
Supplement: Supplementary file 1 — Table S1. Comparison of characteristics between excluded and included participants (n = 1152). (DOC 63 kb) [file 12889_2019_6736_MOESM1_ESM.doc]

**Table S1.** Comparison of characteristics between excluded and included participants (n=1152)

| Characteristics | Excluded  (n=167) | |  | Included  (n=985) | | *P*^a^ |
| --- | --- | --- | --- | --- | --- | --- |
|  | n | % |  | n | % |  |
| Age, n (%) |  |  |  |  |  | <0.001 |
| <25 years | 33 | 19.8 |  | 126 | 12.8 |  |
| 25-34 years | 112 | 67.1 |  | 643 | 65.3 |  |
| ≥35 years | 22 | 13.1 |  | 216 | 21.9 |  |
| Body mass index, n (%) |  |  |  |  |  | 0.771 |
| <23 kg/m^2^ | 92 | 55.1 |  | 531 | 53.9 |  |
| ≥23 kg/m^2^ | 75 | 44.9 |  | 454 | 46.1 |  |
| Ethnicity, n (%) |  |  |  |  |  | 0.311 |
| Chinese | 90 | 53.9 |  | 536 | 54.4 |  |
| Malay | 52 | 31.1 |  | 262 | 26.6 |  |
| Indian | 25 | 15.0 |  | 187 | 19.0 |  |
| Education, n (%) |  |  |  |  |  | 0.003 |
| None/ Primary/ Secondary | 129 | 77.2 |  | 653 | 66.3 |  |
| University | 38 | 22.8 |  | 332 | 33.7 |  |
| Parity, n (%) |  |  |  |  |  | 0.108 |
| Nulliparous | 80 | 47.9 |  | 419 | 42.5 |  |
| Multiparous | 87 | 52.1 |  | 566 | 57.5 |  |
| Smoking status, n (%) |  |  |  |  |  | 0.107 |
| No | 160 | 95.8 |  | 960 | 97.5 |  |
| Yes | 7 | 4.2 |  | 25 | 2.5 |  |
| Iron-containing supplementation, n (%) |  |  |  |  |  | 0.429 |
| No | 22 | 13.2 |  | 123 | 12.5 |  |
| Yes | 145 | 86.8 |  | 862 | 87.5 |  |
| History of anaemia, n (%) |  |  |  |  |  | 0.174 |
| No | 155 | 92.8 |  | 934 | 94.8 |  |
| Yes | 12 | 7.2 |  | 51 | 5.2 |  |

^a^*P* values are determined from Chi-square test.
