# Supplementary material for: Iron status and risk factors of iron deficiency among pregnant women in Singapore: a cross-sectional study
Source: BMC Public Health. 2019 Apr 11;19:397. doi: 10.1186/s12889-019-6736-y (PMC6460529; doi:10.1186/s12889-019-6736-y)
Supplement: Supplementary file 2 — Table S2. Factors associated with iron status during pregnancy assessed by ordinal logistic regression analyses (n = 871). (DOC 63 kb) [file 12889_2019_6736_MOESM2_ESM.doc]

**Table S2.** Factors associated with iron status during pregnancy assessed by ordinal logistic regression analyses (n=871)

|  | Univariable | | |  | Multivariable | | |
| --- | --- | --- | --- | --- | --- | --- | --- |
| Characteristics | Ordinal OR^a^ | 95% CI | *P* |  | Ordinal OR^a^ | 95% CI | *P* |
| Age |  |  |  |  |  |  |  |
| <25 years | 1.61 | 0.83, 3.15 | 0.160 |  | 1.90 | 0.95, 3.80 | 0.068 |
| 25-34 years | 1.26 | 0.87, 1.81 | 0.221 |  | 1.21 | 0.83, 1.76 | 0.325 |
| ≥35 years | Reference |  |  |  | Reference |  |  |
| Body mass index |  |  |  |  |  |  |  |
| <23 kg/m^2^ | Reference |  |  |  | Reference |  |  |
| ≥23 kg/m^2^ | 1.09 | 0.78, 1.53 | 0.608 |  | 0.95 | 0.67, 1.33 | 0.752 |
| Ethnicity |  |  |  |  |  |  |  |
| Chinese | Reference |  |  |  | Reference |  |  |
| Malay | 1.85 | 1.16, 2.95 | 0.010 |  | 2.10 | 1.31, 3.39 | 0.002 |
| Indian | 2.06 | 1.15, 3.69 | 0.015 |  | 1.92 | 1.09, 3.38 | 0.024 |
| Education |  |  |  |  |  |  |  |
| None/ Primary/ Secondary | Reference |  |  |  | Reference |  |  |
| University | 1.43 | 0.97, 2.10 | 0.070 |  | 1.70 | 1.16, 2.51 | 0.007 |
| Parity |  |  |  |  |  |  |  |
| Nulliparous | Reference |  |  |  | Reference |  |  |
| Multiparous | 1.45 | 1.04, 2.02 | 0.029 |  | 1.67 | 1.17, 2.38 | 0.005 |
| Smoking status |  |  |  |  |  |  |  |
| No | Reference |  |  |  | Reference |  |  |
| Yes | 0.71 | 0.30, 1.66 | 0.429 |  | 0.64 | 0.27, 1.53 | 0.316 |
| Iron-containing supplementation |  |  |  |  |  |  |  |
| Yes | Reference |  |  |  | Reference |  |  |
| No | 3.29 | 1.21, 8.98 | 0.020 |  | 2.96 | 1.21, 7.26 | 0.018 |
| History of anemia |  |  |  |  |  |  |  |
| No | Reference |  |  |  | Reference |  |  |
| Yes | 0.96 | 0.47, 1.95 | 0.902 |  | 0.87 | 0.42, 1.79 | 0.698 |

OR, Odds ratio; CI, confidence interval. Iron status was defined according to three ordinal categories of plasma ferritin concentrations: ≥30μg/L (iron sufficiency), 15 to <30μg/L (modest iron depletion) and <15μg/L (severe iron depletion).

^a^Proportional odds ratios with iron sufficiency as the base level, for severe iron depletion versus modest iron depletion/ iron sufficiency, and severe/ modest iron depletion versus iron sufficiency
